# Surgical repair via submammary thoracotomy, right axillary thoracotomy and median sternotomy for ventricular septal defects

**DOI:** 10.1186/s13019-018-0734-5

**Published:** 2018-05-21

**Authors:** Zhi-Nuan Hong, Qiang Chen, Ze-Wei Lin, Gui-Can Zhang, Liang-Wan Chen, Qi-Liang Zhang, Hua Cao

**Affiliations:** Department of Cardiovascular Surgery, Union Hospital, Fujian Medical University, Fuzhou, 350001 People’s Republic of China

**Keywords:** Congenital heart diseases, Ventricular septal defect, Surgery, Submammary thoracotomy, Right axillary thoracotomy, Median sternotomy

## Abstract

**Background:**

Right submammary thoracotomy and right vertical infra-axillary thoracotomy are performed for ventricular septal defect (VSD) to reduce the invasiveness of the conventional surgical repair through median sternotomy approach. No comparative studies have been conducted among these three procedures.

**Methods:**

From January 2016 to December 2016, 182 patients with isolated VSD who underwent surgical repair via one of these 3 approaches were reviewed to compare these three procedures.

**Results:**

The procedure success rates were similar in these three groups. There was no statistically significant difference in operative time, aortic cross-clamping time, the duration of CPB, blood transfusion amount and medical cost. However, postoperative mechanical ventilation time, the duration of intensive care and postoperative length of hospital stay were longer in median sternotomy group than the other two groups. (*P* < 0.05) The median sternotomy group required the longest incision. No significant difference was noted in major adverse events. There were different advantages and disadvantages in the three kinds of operative procedures.

**Conclusions:**

Regarding conventional surgical repair VSD, right submammary thoracotomy and right vertical infra-axillary thoracotomy both delivered better cosmetic results for patients with isolated VSD, while all the three procedures could obtain satisfactory clinical effect.

## Background

Median sternotomy is still considered the gold standard surgical repair approach for ventricular septal defect(VSD) for its high success rate. However, conventional surgical repair can’t avoid long midline scar, long postoperative hospital stay and thoracic deformation. With the development of surgical technique and accumulation of experience, the mortality rate of surgical closure isolated VSD is down to nearly 0 % in recent years. Cosmetic result has become an important factor to evaluate surgical procedures. Thoracoscopic assisted surgery, robotic assisted surgery and different approaches including lower ministernotomy, right submammary thoracotomy, right axillary thoracotomy and right posterolateral thoracotomy have been developed to achieve better cosmetic results. In our institution, we apply right submammary thoracotomy and right subaxillary thoracotomy in patients with isolated VSD, and by document retrieval we found that comparative studies on these three procedures were scarce. In this article, we compare the early and mid-term results of these three procedures.

## Methods

In this study, we reviewed the medical records of 182 patients who underwent VSD closure at our cardiac center between January and December 2016. There were 56 patients in group A (via median sternotomy), 62 patients in group B (via right submammary thoracotomy) and 64 patients in group C (via right vertical infra-axillary thoracotomy). All the patients’ clinical data are shown in Table 1. There were no statistically significant differences in gender, age (median and range, group A: 1.3 years, 2 months-2.5 years, group B: 1.4 years, 6 months-3.0 years, group C: 1.3 years, 6 months-2.8 years), body weight(group A: 6.0 kg, 5.0-12.6 kg, group B: 7.2 kg, 6.2-13.2 kg, group C: 7.3 kg, 5.9-13.0 kg) or VSD size(group A: 6.0 mm, 3.0-8.2 mm, group B: 5.4 mm, 3.1-7.8 mm, group C: 5.2 mm, 3.0-7.6 mm) distribution among these three groups. Routine clinical examinations were performed, which included a standard lead electrocardiogram, a chest X-ray examination, and routine blood and biochemical tests. All the patients had a confirmed diagnosis of VSD and were sufficiently assessed by transthoracic echocardiography(TTE). Pulmonary hypertension (which was accessed by TTE) (group A: 60 mmHg, 30-82 mmHg, group B: 54 mmHg, 32-78 mmHg, group C: 52 mmHg, 30-76 mmHg) and cardiothoracic ratio (group A: 0.58, 0.40-0.72, group B: 0.56, 0.42-0.61, group C: 0.57, 0.47--0.61) were similar among these three groups. The inclusion criteria: isolated VSD and no other intracardiac malformation. The exclusion criteria: respiratory diseases, history of thorax procedure, history of failed attempt of transcatheter closure, severe valvular regurgitation, and right to left shunt caused by severe pulmonary hypertension. The successful VSD closure were defined as no large residual shunt (< 2 mm) assessed by postoperative TTE.

**Table 1:** Preoperative data comparison among three groups of patients

Item	Group A	Group B	Group C	*P*
*N*	56	62	64	
Age (year)	1.1 ± 1.4	1.3 ± 1.2	1.3 ± 1.5	*P* > 0.05
Gender (M/F)	26/30	31/31	30/34	*P* > 0.05
Weight (kg)	6.1 ± 3.2	8.3 ± 2.3	8.4 ± 2.5	*P* > 0.05
Size of VSD (mm)	6.2 ± 1.05	5.3 ± 1.12	5.1 ± 1.04	*P* > 0.05
Perimembrous VSD	48	60	63	
Muscular VSD	1	0	0	
Subatrial VSD	7	2	1	
Pulmonary hypertension (mmhg)	45.2 ± 11.7	42.1 ± 8.2	40.5 ± 9.2	*P* > 0.05
Cardiothoracic ratio	0.61 ± 0.10	0.56 ± 0.08	0.57 ± 0.06	*P* > 0.05

### Operative technique

#### VSD surgical repair via median sternotomy (group A)

Conventional surgical closure was conducted through median sternotomy approach under cardiopulmonary bypass. Pericardial patch was conducted in all patients.

#### VSD surgical repair via right submammary thoracotomy (group B)

The patients were canted to the right at an angle of 20° to 30°, with the right arm positioned beside the body. Under general anesthesia, we performed the incision in the right submammary groove, while with the cases of undeveloped breasts we made the incision in the fourth intercostal space to avoid deformity of the breast and the pectoral muscle. Then we spread muscle softly to avoid causing trauma to the rib periosteum and try to preserve the right internal mammary vessels [1]. The incision was about 4-5 cm based on the patient’s height and weight. To provide a better operation field, we used a small rib spreader. The pericardium was opened in the site 1 cm anterior to the phrenic nerve and suspended to expose the right atrium and the aortic root.

Then we performed cannulations of the ascending aorta, the superior vena cava and the inferior vena cava to setup a standard CPB after systemic heparin administration. Aortic cross-clamp was performed in the same incision, then isothermal blood cardioplegia was provided to achieve adequate myocardial protection. To get enough exposure of the intracardiac anatomy, we performed a standard oblique right atriotomy, and did the procedures for VSD surgical closure as used in the median sternotomy approach. Finally, we gradually discontinued the CPB and partially closed the pericardium. We closed the chest in a routine fashion with an intradermal continuous suture for the skin after placement of chest drainage.

#### VSD surgical repair via right vertical infra-axillary thoracotomy (group C)

The patient was canted to the right at an angle of 90° with the right arm placed on the head and the shoulder-joint abducted at approximately 120°. The right vertical infra-axillary skin incision was done from the second intercostal space along the right midaxillary line to the fifth intercostal space along the preaxillary line. The incision was about 4-5 cm, adjusted to patient’s weight and height. We entered the right thoracic cavity through the third or fourth intercostal space after muscle sparing and retracted the lung posteriorly with a wet gauge to expose the pericardium. The pericardium, opened from 2 cm superior to the phrenic nerve, was then suspended with sling sutures at the superior, middle, and inferior aspects of the pericardium to expose the right atrium and the aortic root.

We placed standard purse sutures in right side of the ascending aorta, the superior vena cava, and the right atriocaval junction. After heparinization, we cannulated the ascending aorta, the superior vena cava and the inferior vena cava. Then, a standard CPB were started and aortic cross-clamp was performed in the same incision. The same VSD closure was done as used in other procedures. Finally, we closed the pericardium partially and place a chest drainage in the right thorax.

All patients were given TTE, electrocardiogram, physical examinations 3 months, 6 months and 1 year after their operations.

### Statistical analysis

Continuous variables were expressed as x ± s in table, t-test or analysis of variances were applied to continuous variables and the χ2 or Fisher’s test to categorical variables. *P* value< 0.05 were defined as statistical significance.

## Results

The procedures were successful in the patients of these three groups. No perioperative death, low cardiac output syndrome, malignant arrhythmia, delayed recovery, re-operation for VSD, complete atrioventricular block (AVB), or cerebrovascular events were recorded in any group. Comparison among these three groups, shows that there were no statistically significant differences in operative time, aortic cross-clamping time, duration of CPB, or blood transfusion amount. (*P* > 0.05).

However, postoperative mechanical ventilation time (group A: 16.0 h, 8.8-22.0 h, group B: 8.0 h, 6.3-17.2 h, group C: 7.8 h, 5.0-16.4 h), duration of intensive care (group A: 20.0 h, 16.5-24.3 h, group B: 13. 6 h, 10.0-16.2 h, group C: 13.5 h, 9.8-17.6 h), postoperative length of hospital stay (group A: 9.0 days, 6.2-15.3 days, group B: 6.5 days, 3.2-9.0 days, group C: 6.4 days, 3.4-11.0 days), and pleural fluid drainage (group A: 50.0 ml, 20.0-100.5 ml, group B: 22.0 ml, 10.5-45.5 ml, group C: 20.5 ml, 8.5-47.5 ml) in group A was larger than that in group B and C (*P* < 0.05). Medical cost was similar among the three groups (*P* > 0.05). (Table 2) Group A required the longest incision (13.0 cm, 11.2-15.0 cm). The incidence rates of postoperative pulmonary infection were not significantly different, but the incidence rates of postoperative pneumothorax and subcutaneous emphysema were significantly higher in group B and C (*P* < 0.05). (Table 3).

**Table 2:** Peri-operative and post-operative data comparison among three groups of patients

Item	Group A	Group B	Group C	*P*
Operative time (min)	110.4 ± 22.3	105.2 ± 25.4	103.6 ± 18.5	*P* > 0.05
Aortic occlusion clamping time (min)	38.3 ± 11.5	36.4 ± 12.8	37.1 ± 9.9	*P* > 0.05
Cardiopulmonary bypassing time (min)	60.5 ± 12.3	62.5 ± 10.2	63.2 ± 13.6	*P* > 0.05
Mechanical ventilation time (h)	16.6 ± 4.4	10.4 ± 3.1*	9.8 ± 2.5*	*P* < 0.05
Intensive care unit time (h)	21.7 ± 5.2	14.5 ± 2.3*	13.8 ± 2.6*	*P* < 0.05
Drainage (ml)	50.5 ± 21.5	24.4 ± 15.6*	20.5 ± 18.6*	*P* < 0.05
Blood transfusion volume (ml)	320.5 ± 80.4	338.3 ± 96.7	340.5 ± 86.5	*P* > 0.05
The incision length (cm)	13.7 ± 1.1	10.2 ± 2.3*	8.5 ± 2.4*	*P* < 0.05
Postoperative hospital stay (d)	9.2 ± 3.2	6.8 ± 2.1*	6.9 ± 3.2*	*P* < 0.05
Hospital costs (10000RMB)	5.85 ± 0.63	5.45 ± 0.83	5.25 ± 0.92	*P* > 0.05

**Table 3:** Post-operative complications comparison between two group of patients(%)

Item	Group A	Group B	Group C	*P*
Cerebrovascular accident	0	0	0	
Small residual shunt	5	3	4	
Large residual shunt requiring reoperation	0	0	0	
Severe Arrhythmia				
Complete AVB	0	0	0	
Mobitz type II AVB	0	0	0	
Low cardiac output syndrome	0	0	0	
Pulmonary infection	5	7	9	*P* > 0.05
Surgical wound bad healing	4	0	1	
Pneumothorax	0	3	4	*P* < 0.05
Subcutaneous emphysema	0	2	3	*P* < 0.05
Thoracic deformity	7	0	0	
Pericardial effusion	2	0	0	
Pleural effusion	0	2	2	
Transient Arrhythmia	5	6	5	

During postoperative follow-ups, no serious complications or malignant arrhythmia occurred in any of these patients. Through physical examinations, we found 7 cases with thoracic deformity in group A. Then, these 7 patients were given thoracic CT scan and the results showed 5 cases with pectus carinatum and 2 cases with funnel chest. The above 7 patients were asymptomatic during the follow-up period. So close medical observation were given to these 7 patients without any intervention. No chest deformity were found in the other two groups. CT Haller index changes in the above 7 patients were shown in Table 4. No significant progress of these patients was found in the follow-up period.

**Table 4:** CT Haller Index change in 7 patients with thoracic deformity

CT Haller Index	3-month	6-month	12-month	*P*
Pectus Carinatum (*n* = 5)	2.30 ± 0.19	2.31 ± 0.20	2.31 ± 0.18	*P* > 0.05
Funnel Chest (*n* = 2)	3.00 ± 0.03	3.00 ± 0.03	3.00 ± 0.03	*P* > 0.05

## Discussion

Ventricular septal defect is one of the most common congenital cardiac defects [1, 2]. Although the transcatheter techniques have been widely used and performed and delivered promising early and midterm results in recent years, the X-ray exposure and potential vascular injury limited the promotion of this approach [3–5].And the median sternotomy approach was limited by its visible mid-sternotomy scar, which should be taken into consideration. Surgeons then developed other approaches to reduce the invasiveness of complete median sternotomy and pursue better cosmetic results, especially in children, teenager and female patients [6]. These alternatives included lower mini-sternotomy incision [7, 8], right submammary incision [9, 10], right posterolateral thoracotomy incision [11] and right vertical infra-axillary incision [12–14].

In our institution, we applied right submammary thoracotomy and right vertical infra-axillary thoracotomy in VSD repair. The advantages of the right submammary thoracotomy and right vertical infra-axillary thoracotomy have been previously described. These two thoracotomies are similar to median sternotomy in having enough exposure, no need for special instrument, no difficulty in CPB establishment, and no increased medical cost in most reports. And their advantages have been described, such as faster recovery and better cosmetic results. However, technical complexity including satisfactory exposure, invisible site and the length of incision remained the focus of exploration. As no comparative study has been conducted among right submammary thoracotomy, right vertical infra-axillary thoracotomy and median sternotomy, our study aims to compare these three approaches in repairing VSD under CPB.

Compared with group A, there was no significant difference in CPB time, aortic cross-clap time or operative time in group B and C. We contributed these to those right submammary thoracotomy and right vertical infra-axillary thoracotomy could provide enough good exposure of the inferior vena cava and the ascending aorta without increasing the technical difficulty. However, according to our experience, it’s difficult to setup CPB in female patients with large breast and in patients with a BMI > 28 kg/m [2] in group B and C. The chest incision in these two groups were also significantly shorter than the median sternotomy group. Meanwhile, there was no need for sternum split or wire fixation, which may lead to negative post-operative X-ray chest examination. But the incidence rates of postoperative pneumothorax and subcutaneous emphysema were significantly higher in group B and C. With strengthened operative technique and management, however, such complications can be well controlled. The surgical success rate of VSD repair via right submammary thoracotomy and right vertical infra-axillary thoracotomy were similar to median sternotomy in this study, which means all the three methods can achieve satisfactory clinical results. Based on these findings, we recommend that right submammary thoracotomy and right vertical infra-axillary thoracotomy be used as effective and safe alternatives in VSD surgical repair.

Cosmetic results should be taken into consideration in such operations, which include incision length, visible or invisible and whether this incision causes thoracic deformity. We found that the incidence of thoracic deformity was higher in median sternotomy group, although 7 patients suffered from pectus carinatum or funnel chest. No significant progress was found in the follow-up period. Close medical observation were given to these 7 patients without any other intervention. In most medical centers, the right submammary thoracotomy was made through the fourth or fifth intercostal space. Compared to the median sternotomy group, the incision caused can’t be seen from the collar, which may be more acceptable to women patients. However, this incision may dissect the breast tissue and result in asymmetrical development and a decrease in sensitivity of the nipple [15, 16]. We contributed these side effects to the difference in incision choice in our institution, where the incision was in the fourth intercostal space for children. In addition, we also obey the rule that the incision should be at least 1.5 cm away from the mammary areola in patients with undeveloped breasts [17]. For female patients the incision was in the submammary groove so that when the operation via the incision would be covered by breast. In addition, during the procedure, we did our best to preserve the right internal mammary vessels.

In our institution, we also repaired VSD via right vertical infra-axillary thoracotomy. Wang et al. reported 274 patients with ventricular septal defects went through repair via a minimal right vertical infra-axillary thoracotomy [14]. In their study there were no deaths or complications from the infection of incisional wound and arrhythmia, and no significant differences in CPB time or postoperative ventilator time. However, the length of incision, postoperative volume drainage and ICU stay, minimal right vertical infra-axillary thoracotomy was significantly shorter than median sternotomy. These results were consistent with the results of our study. First, unlike some other new technology, this incision didn’t require special instruments and most part of the procedure is similar to conventional surgical repair, which means no increase in hospital cost and short learning cure for experienced surgeons. Second, it could provide enough surgical field. Based on these two reasons, the CPB time and the aortic clamping time of this procedure were also similar to those of median sternotomy. And we also found that the hospital stay and the time needed to recover to normal activities in the right vertical infra-axillary thoracotomy group were superior to those of the median sternotomy group, which also suggested faster recovery. Although in most patients this procedure didn’t increase the technical difficulty, it’s still difficult, even for experienced surgeons, to close subarterial VSDs through such procedure. In repairing such kind of VSD, we preferred the right submammary thoracotomy to right vertical infra-axillary thoracotomy, which also provide enough surgical field if placing a wet sponge under pericardial cavity beneath the heart.

For female patients with developed breast, if we chose right submammary thoracotomy, the incision would be covered by breast. However, when operating on children, right vertical infra-axillary thoracotomy may be a better choice. The reasons are listed below: First, this short incision is located under the armpit, which makes it almost invisible; Second, the incision of right submammary thoracotomy never surpasses the preaxillary line and thus will not cause dysplasia. Finally, the incision site is in the chest wall and far from the costochondral junction. Thus, it does not interfere with the development of the chest wall.

The incidence of arrhythmia was similar in all groups. According to previous report, surgical approach (right atrium or right ventricle) did not influence the arrhythmia rate [18, 19], which was the reason why we didn’t conduct subgroup analysis of arrhythmia. According to above-mentioned comparisons, we could conclude that right submammary thoracotomy and right vertical infra-axillary thoracotomy are both safer and more efficient approaches for VSD closure than median sternotomy. We then further compared right submammary thoracotomy and right vertical infra-axillary thoracotomy. We found that there weren’t significant differences in procedure success rate, operation time, ICU stay, postoperative hospital stay or volume of transfusion in these two groups. And the rate of complications were also similar in right submammary thoracotomy and right vertical infra-axillary thoracotomy.

This study was a retrospective study and was limited by the number of cases and the fact that it was done in a single center. Prospective randomized controlled studies with a larger sample size, even multi-center cooperation, must be conducted to confirm the results. In addition, a longer follow-up is essential, especially for those patients with thoracic deformity.

## Conclusion

All the three procedures can obtain satisfactory clinical effects. Regarding surgical repair VSD, right submammary thoracotomy and right vertical infra-axillary thoracotomy both deliver good cosmetic results for patients with isolated VSD, while the latter may be a better choice to pursue better cosmetic results for children.

## Abbreviations

AVB: atrioventricular block
CHD: congenital heart disease
ICU: intensive care unit
TTE: transthoracic echocardiography
VSD: ventricular septal defect
